# Supplementary material for: Genomic and Functional Variation of the Chlorophyll d-Producing Cyanobacterium Acaryochloris marina
Source: Microorganisms. 2022 Mar 6;10(3):569. doi: 10.3390/microorganisms10030569 (PMC8949462; doi:10.3390/microorganisms10030569)

Table S1. Sampling information for *A. marina* strain collection.

Strain	Location	Sampling info	Collection Date
S1	Shelter Cove, CA	Upper intertidal pool, from surface of *Mastocarpus papillatus*	7/20/16
S7	Shelter Cove, CA	Surface of *Corallina* sp.	7/20/16
S9	Shelter Cove, CA	Surface of *Schizymenia pacifica*	7/20/16
S15	Shelter Cove, CA	Surface of *Pikea pinnata*	7/20/16
HP1	Hug Point State Park, OR	Algae on upper intertidal rocks	6/28/17
HP3	Hug Point State Park, OR	Algae on upper intertidal rocks	6/28/17
HP5	Hug Point State Park, OR	Algae on upper intertidal rocks	6/28/17
HP8	Hug Point State Park, OR	Algae on upper intertidal rocks	6/28/17
HP9	Hug Point State Park, OR	Algae on upper intertidal rocks	6/28/17
HP10	Hug Point State Park, OR	Algae on upper intertidal rocks	6/28/17
HP11	Hug Point State Park, OR	Algae on upper intertidal rocks	6/28/17
MSP2	MacKerricher State Park, CA	Surface of coralline red alga	5/28/18
GR1	Glover's Reef, Belize	Red algae from floating lagoon mat	1/16/18
Awaji (MU01)	Esaki, Awajishima, Hyogo, Japan	Epiphyte of red alga, *Ahnfeltiopsis flabelliformis*	2001
MU03	Muroran, Hokkaido, Japan	Epiphyte of red alga, *Ahnfeltiopsis flabelliformis*	3/7/06
MU04	Muroran, Hokkaido, Japan	Endophyte of red alga, *Ahnfeltiopsis flabelliformis*	3/7/06
MU05	Arabian Sea	Epiphyte of red alga, *Gelidium* sp.	5/3/06
MU06	Tanoshiro, Awajishima, Hyogo, Japan	Epiphyte of red alga, *Callophyllis japonica*	6/9/06
MU07	Yura, Awajishima, Hyogo, Japan	Epiphyte of red alga, *Callophyllis japonica*	8/10/06
MU08	South China Sea	Epiphyte of red alga (unidentified)	1/8/07
MU09	Enoshima, Kanagawa, Japan	Epiphyte of red alga, *Ahnfeltiopsis flabelliformis*	2/3/07
MU10	Gushikami, Okinawa, Japan	Epiphyte of red alga (unidentified)	2/7/07
MU11	Bise, Okinawa, Japan	Isolate from tunicate, *Diplosoma virens*	4/3/07
MU12	Bise, Okinawa, Japan	Isolate from tunicate, *Trididemnum clinides*	4/3/07
MU13	Kurimajima, Okinawa, Japan	Isolate from tunicate, *Lissoclinum punctatum*	12/10/08
NIES2412	Muroran, Hokkaido, Japan	Seaweed (unidentified)	6/20/04
CCMEE 5410	Salton Sea, CA	Epilithic microbial mat	1999
MBIC11017	Republic of Palau coastline	Surface of colonial ascidian, *Lissoclinum patella*	1996
P4	Praia de Carcavelos, Portugal	Algae from intertidal rocks	10/24/18
P9	Praia de Carcavelos, Portugal	Algae from intertidal rocks	10/24/18
FH1	Friday Harbor, San Juan Island, WA	Algal samples from dock tires	1/5/19
FH2	Friday Harbor, San Juan Island, WA	Algal samples from dock tires	1/5/19

FH6	Friday Harbor, San Juan Island, WA	Algal samples from dock tires	1/5/19
FH11	Friday Harbor, San Juan Island, WA	Algal samples from dock tires	1/5/19
WB4	Wreck Beach, Vancouver, BC	Red alga on sand flat at low tide	8/7/18
I2_1	Queroianella, Italy	Red alga on rock	12/30/19

Table S2. Genome assembly statistics.

Strain	N50	# contigs	Estimated genome size	Completeness (%) [a]	BUSCO (%)	Reference
S1	61925	398	7345427	99.29	98.9	Ulrich *et al*. 2021
S7	54529	446	7118253	99.53	99	Ulrich *et al*. 2021
S9	58396	410	7273775	99.29	98.9	Ulrich *et al*. 2021
S15	5881945	7	7112772	99.53	99	Ulrich *et al*. 2021
HP1	36836	890	7392918	99.29	98.9	Ulrich *et al*. 2021
HP3	47151	516	7745077	99.53	99	This study
HP5	63851	422	7509040	99.53	98.7	This study
HP6	89219	374	7275896	99.53	99	Ulrich *et al*. 2021
HP8	39624	1098	7987243	99.29	99	Ulrich *et al*. 2021
HP9	36507	820	7003118	99.53	98.8	Ulrich *et al*. 2021
HP10	37829	1214	7998694	99.29	99	Ulrich *et al*. 2021
HP11	58563	573	7890141	99.53	99	This study
MSP2	48560	761	7090956	99.29	98.9	Ulrich *et al*. 2021
GR1	41795	470	6175695	99.53	97.8	Ulrich *et al*. 2021
Awaji	48724	758	7664765	99.29	98.7	Ulrich *et al*. 2021
MU03	72346	458	6106866	99.53	98.8	Ulrich *et al*. 2021
MU04	43413	616	6967674	99.53	98.9	Ulrich *et al*. 2021
MU05	102057	138	5973023	96.7	96.5	Ulrich *et al*. 2021
MU06	27254	1219	8857603	99.76	98.7	Ulrich *et al*. 2021
MU07	32970	1060	8711329	99.76	99	Ulrich *et al*. 2021
MU08	35868	443	6609766	98.82	98.5	Ulrich *et al*. 2021
MU09	52586	337	6056097	98.58	98.3	Ulrich *et al*. 2021
MU10	64774	293	6553268	99.53	98.7	Ulrich *et al*. 2021
MU11	42252	406	7305935	99.53	98	Ulrich *et al*. 2021
MU12	16188	795	7405669	98.82	93.9	Ulrich *et al*. 2021
MU13	45821	486	7134557	99.53	98.6	Ulrich *et al*. 2021
NIES2412	41849	895	7860937	99.29	98.7	Ulrich *et al*. 2021
CCMEE 5410	4516345	23	8072368	99.53	98.5	Ulrich *et al*. 2021
MBIC11017	6503724	10	8361599	99.53	98.9	Swingley *et al*. 2008
P4	75683	271	6422642	99.53	98.8	This study
P9	68391	364	6939514	99.53	98.9	This study
FH1	45019	760	7870824	99.29	98.7	Ulrich *et al*. 2021
FH2	30273	720	7979362	99.53	98.9	This study
FH6	60621	505	8028323	99.29	98.9	This study
FH11	89858	355	7247103	99.53	99	This study
WB4	39403	290	5820594	99.53	98	This study
I2.1	34011	858	8008360	99.76	98.5	This study

[a] Genome completeness was assessed with CheckM v1.0.18

Figure S1. 16S rRNA gene phylogeny showing HICR111A as a member of the same clade as other nitrogen-fixing strains (GR1, MU08, MU09).

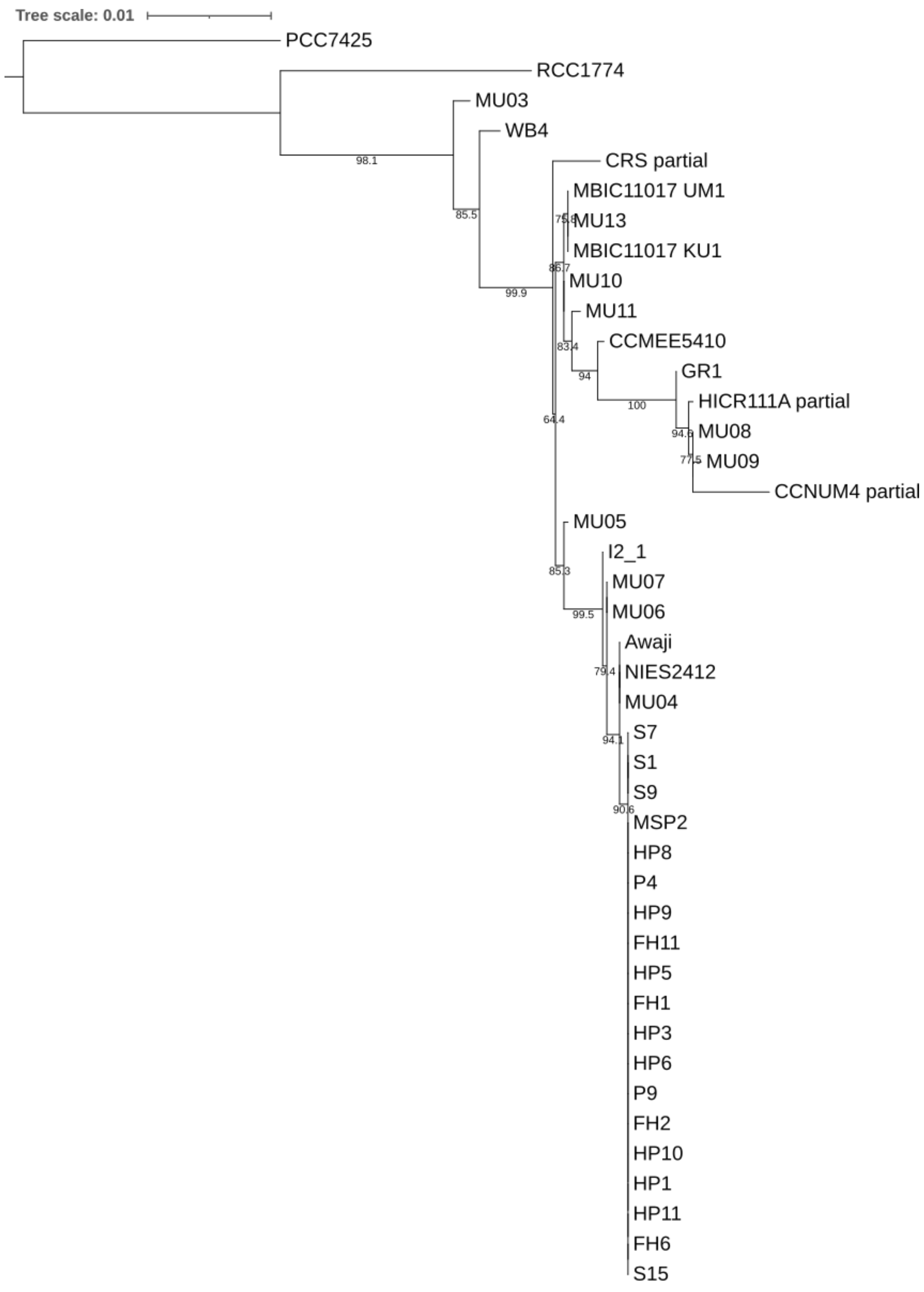

Figure S2. Dendrogram of *A. marina* iron gene content, hierarchically clustered by Euclidean distance. Bootstrap values for each cluster shown in green, and approximately unbiased P-values are shown in red.

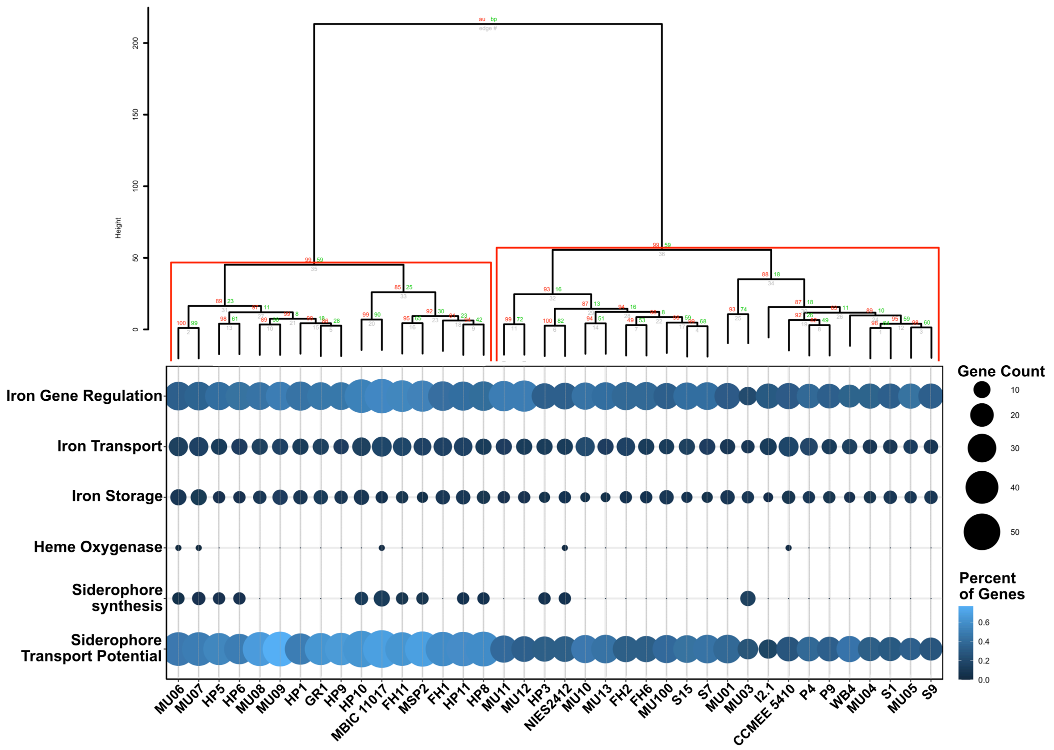

Supplement: Supplementary file 1 [file microorganisms-10-00569-s001.zip › Tables S1 and S2, Figures S1 and S2.pdf]
